# Identification and Validation of a Pyroptosis-Related Prognostic Model for Gastric Cancer

**DOI:** 10.3389/fgene.2021.699503

**Published:** 2022-02-25

**Authors:** Chaowei Liang, Jiaxin Fan, Chaojie Liang, Jiansheng Guo

**Affiliations:** ^1^ Department of Gastrointestinal Surgery, First Hospital of Shanxi Medical University, Taiyuan, China; ^2^ Department of Hepatobiliary and Pancreatic Surgery, First Hospital of Shanxi Medical University, Taiyuan, China

**Keywords:** gastric cancer, pyroptosis, prognostic signature, nomogram, the cancer genome atlas

## Abstract

Pyroptosis is an inflammatory form of programmed cell death triggered by caspase-1/4/5/11 that plays an important role in the occurrence and development of gastric cancer (GC). We investigated the prognostic value of pyroptosis-related genes in GC. The “LIMMA” R package and univariate Cox analysis were used to find pyroptosis-related genes with differential expression and prognostic value in the TCGA cohort and the identified genes were analyzed for GO enrichment and KEGG pathways. The selected genes were then included in a multivariate Cox proportional hazard regression analysis, and a ten genes prognostic model (BIRC2, CD274, IRGM, ANXA2, GBP5, TXNIP, POP1, GBP1, DHX9, and TLR2) was established. To evaluate the predictive value of the risk score on prognosis, patients were divided into high-risk and low-risk groups according to the median risk score, and survival analysis was carried out. Compared with the low-risk group, the OS of GC patients in the high-risk group was significantly worse. Additionally, these results were verified in the GSE84437 and GSE66229 datasets. Finally, through the combination of prognostic gene characteristics and clinicopathological features, a nomogram was established to predict individual survival probability. The results show that the genetic risk characteristics related to clinical features can be used as independent prognostic indicators for patients with GC. In summary, the pyroptosis-related risk signals proposed in this study can potentially predict the prognosis of patients with GC. In addition, we also found significant infiltration of dendritic cells, macrophages, and neutrophils in tissues of high-risk patients.

## Introduction

Gastric cancer (GC) is the fifth most common cancer and the third most common cause of cancer death in the world, with more than one million new gastric cancer patients each year, of which about 73% die. The median survival time of advanced gastric cancer is less than 12 months (Smyth et al., 2020). Gastric cancer is the third most common cancer in Asia, after breast and lung cancer (Sung et al., 2021). The occurrence of gastric cancer is a multi-factor, multi-step process, and a variety of mechanisms affect its occurrence and development (Chia and Tan, 2016), making the prognosis evaluation full of challenges. Therefore, it is very important to find an effective prediction model.

Pyroptosis is an inflammatory form of programmed cell death triggered by caspase-1/4/5/11 (Bergsbaken et al., 2009). Caspase-1 and-11 trigger pyroptosis through the cleavage of Gasdermin D (Kayagaki et al., 2015; Shi et al., 2015). The characteristics of pyroptosis are pore formation, cell swelling, plasma membrane rupture, and release of intracellular contents (Fang et al., 2020). Pyroptosis may affect all stages of cancer development and has therefore become a new topic in cancer research (Ruan et al., 2020). Recent studies have shown that polyphyllin VI induces the transition of A549 and H1299 cells from apoptosis to pyroptosis by activating caspase-1, which induces the ROS/NF-κB/NLRP3/GSDMD signal axis, resulting in cell death (Teng et al., 2020). GSDME can convert TNF-α, chemotherapy, or caspase-3-mediated apoptosis into pyroptosis (Wang et al., 2017; Yu et al., 2019; Wang et al., 2017; Yu et al., 2019). Caspase-3-dependent apoptosis and pyroptosis can promote the clearance of stressed, injured, transformed, or infected cells, which plays a very important role in the development and treatment of tumors (Jiang et al., 2020). PD-L1 converts tumor cell apoptosis induced by TNF-α into pyroptosis, resulting in tumor necrosis (Hou et al., 2020a). Mutant BRAF and MEK inhibitors regulate the tumor immune microenvironment through pyrogenesis (Erkes et al., 2020). Thanks to the existing research results, it is known that pyroptosis is involved in tumorigenesis and anti-tumoral processes, but studies on its specific function in GC are missing. It is not clear whether pyroptosis-related genes are linked to the prognosis of patients with gastric cancer.

We systematically analyzed the differentially expressed genes related to pyroptosis in gastric cancer in TCGA database samples. Univariate analysis was used to screen the genes related to prognosis, and then the resulting genes were analyzed by multivariate Cox proportional hazard regression analysis to establish a prognostic model. A prognostic nomogram containing prognostic gene markers was established to predict overall survival. Since previous studies showed a significant correlation between pyroptosis and the immune microenvironment in GC (Shao et al., 2021), we also studied the infiltration of immune cells in tumor cells.

## Materials and Methods

### Data Acquisition

Transcriptome sequencing data, survival information, and clinical information of TCGA gastric cancer data set obtained were obtained from the TCGA website (https://portal.gdc.cancer.gov/). Two external validation datasets, GSE84437 and GSE66229, were downloaded from the Gene Expression Omnibus database (GEO). The R (version 4.0.2) software was used to standardize and process the data. Pyroptosis-related genes were searched through THE HUMAN GENE DATABASE (https://www.genecards.org/) with the keyword “Pyroptosis.” Therefore, 121 genes related to pyroptosis were included in the analysis and provided in Supplementary Material S1. First of all, the differentially expressed pyroptosis-related genes in TCGA gastric cancer tissues were identified by using the “LIMMA” R software package: false detection rate (FDR) < 0.05.

### GO and KEGG Functional Enrichment

Gene Ontology (GO) enrichment analysis of 51 differentially expressed pyroptosis-related genes was carried out with the R software package “clusterprofiler,” The selected background gene set is compiled by others, which is the human genome annotation in Carlson M’s “org.Hs.eg.db,” including biological process (BP), cellular component (CC), and molecular function (MF). The filter conditions were pvalueCutoff = 0.05, qvalueCutoff = 0.05. The same tool was used to analyze the pathways enrichment according to the Kyoto Encyclopedia of Gene and Genome (KEGG).

### PPI Network

Protein-protein interaction (PPI) networks in differentially expressed pyroptosis-related genes were constructed using the STRING database and visualized with the Cytoscape software. The Molecular Complex Detection (MCODE) of the Cytoscape plugin was used to detect the important modules in the PPI network, and GO and KEGG analyses were carried out to further study its molecular function in gastric cancer.

### Establishment and Verification of Prognostic Model

Univariate Cox proportional hazard regression analysis was used to screen pyroptosis-related genes significantly associated with overall survival (OS) in TCGA gastric cancer data set. Then multivariate Cox proportional hazard regression analysis was performed to establish a prognostic model. We use the following formula to calculate the risk score for each patient: risk score = e^sum (each gene’s expression×corresponding coefficient)^. Patients were divided into high-risk and low-risk groups according to the median value of the risk score.

To determine the role of the risk score in predicting the clinical prognosis of patients with gastric cancer, we analyzed the difference in survival time between the high-risk group and low-risk group by the Kaplan-Meier method. To verify whether our prognostic model is also applicable to other datasets, we selected GSE84437 and GSE66229 gastric cancer datasets from the GEO database for external verification and calculated the risk score using the same formula as the TCGA cohort in these two datasets. Univariate and multivariate Cox regression analyses were used to study whether pyroptosis-related risk index could be an independent predictor of OS in the TCGA dataset of patients with gastric cancer. Risk score, age, sex, tumor subtype, pathological stage, and histological grade were regarded as covariates.

Copy number changes and mutations of key genes were investigated using the online database cBioPortal (Gao et al., 2013), and protein expression data were retrieved from the human protein map (HPA) database.

### The Construction of Nomogram

According to age, staging, grading, T, N, M, and risk score, the nomogram was constructed with “RMS” and “survival” software packages in R. The evaluation of the consistency between actual and predicted survival is achieved by generating a calibration curve. Finally, the ROC curve of the nomogram changing with time is generated, and the AUC value is calculated.

### Analysis of the Correlation Between Risk Score Model and Immune Cell Infiltration

We used tumor immune estimation resources (TIMER), which is a reliable resource for comprehensive analysis of tumor-infiltrating immune cells, to explore the relationship between prognostic models and immune cell infiltration. The TIMER algorithm can help users estimate the composition of six tumor-infiltrating immune cell subpopulations (B cells, CD4+T cells, CD8+T cells, macrophages, neutrophils, and dendritic cells). The level of immune infiltration in patients with gastric cancer was obtained from the TIMER website (http://cistrome.dfci.harvard.edu/TIMER/), and the correlation between 6 tumor-infiltrating immune cell types and our prognostic model was analyzed in R.

### Statistical Analysis

**FIGURE 1:**
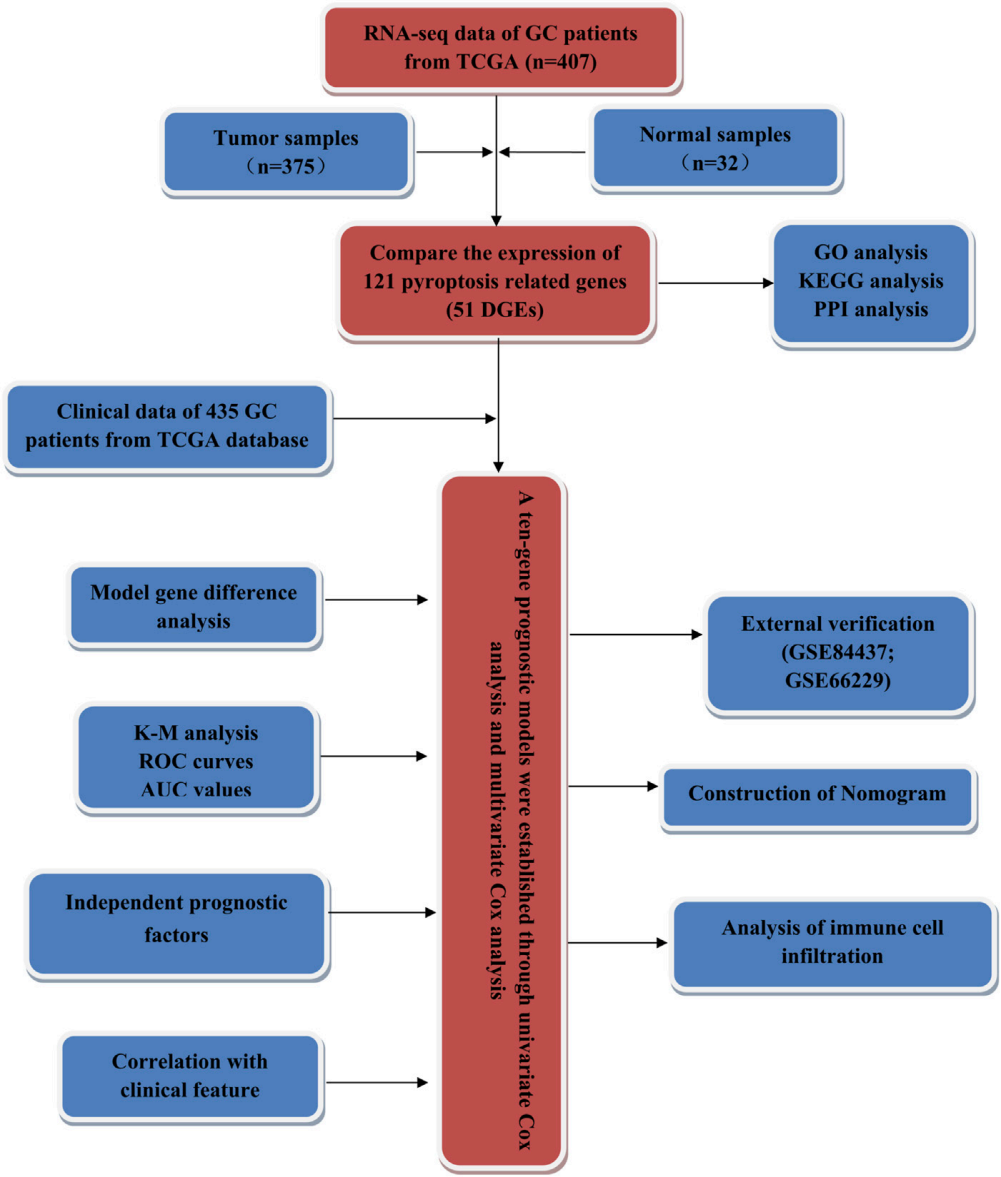
The flowchart of this study.

Further statistical analyses were performed with the R software (v4.0.2). Wilcoxon ranked sum (Mann Whitney) test was used to screen the differentially expressed pyroptosis-related genes between tumor and adjacent tissues. The independent predictors of OS and the relationship between risk score and clinical information and prognosis were determined by univariate and multivariate Cox regression analysis. The difference of OS between the high-risk group and low-risk group was compared by the Kaplan-Meier method, and the *p*-value was calculated by logarithmic rank-sum test. T-test was used to compare the differences in risk scores among different clinical feature groups. *p* < 0.05 was considered to be statistically significant. We conducted our study as described in the flowchart (Figure 1).

## Result

### Identification and Enrichment Analysis of Differentially Expressed Genes

**FIGURE 2:**
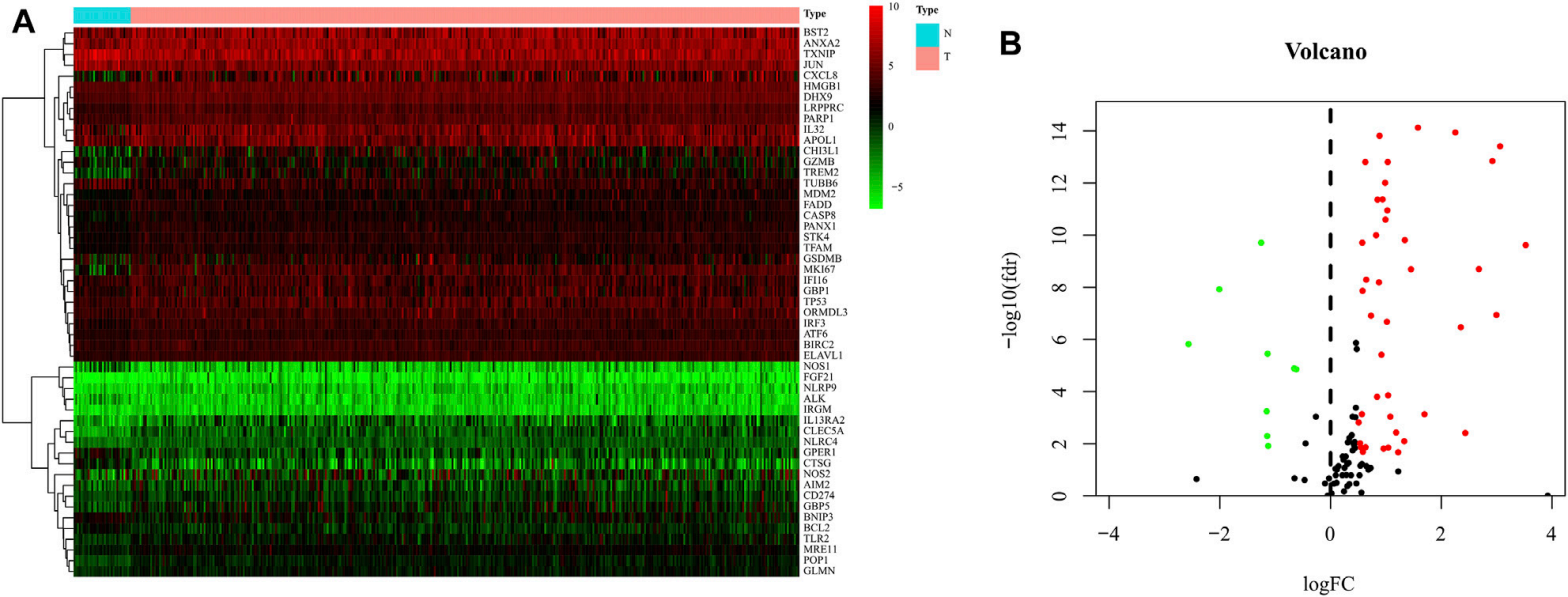
Identification of differential expressed genes (DEGs) in GC and normal tissues. **(A)** Heat map of 51 DEGs in TCGA. Red: upregulation; Green: downregulation. The abscissa represents the type, N normal; T Tumor; ordinate represents the gene. **(B)** Volcano plots of the distributions of 51 DEGs. The abscissa represents logFC and the ordinate represents -log10 (FDR).

**FIGURE 3:**
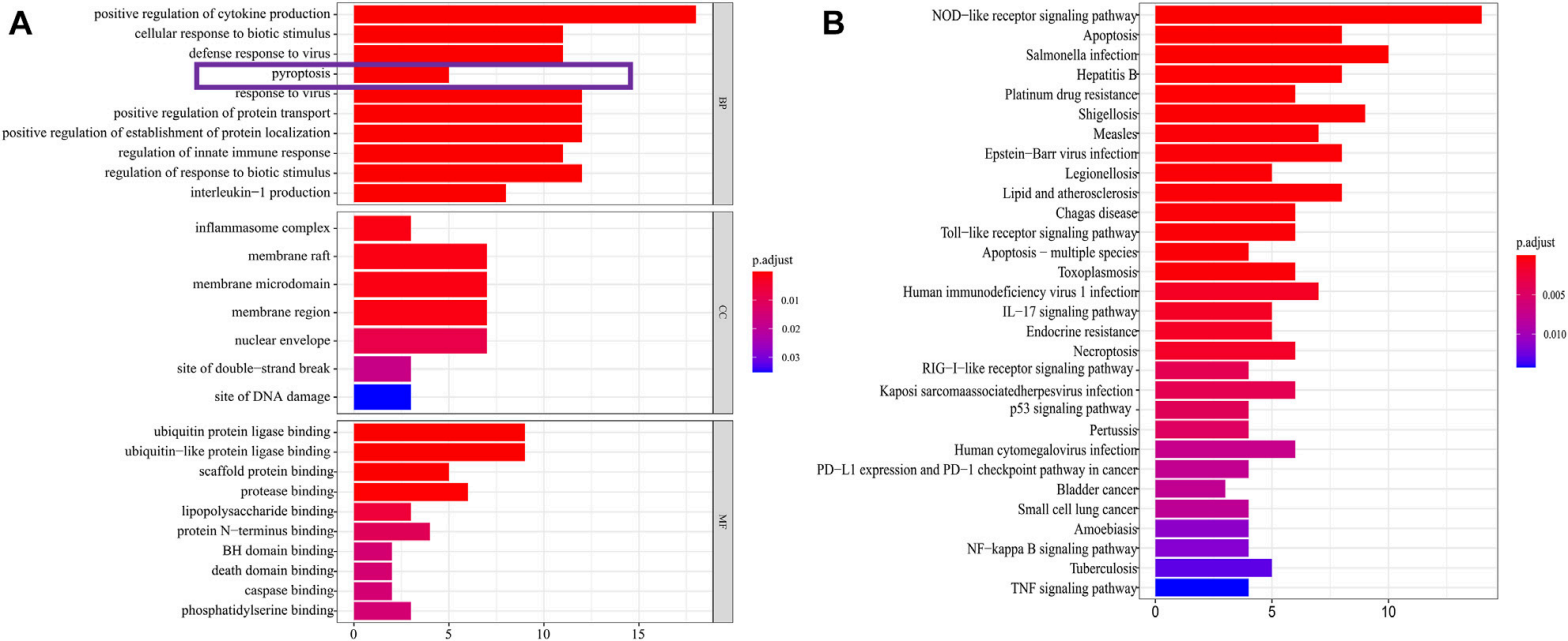
DEGs enrichment analysis. **(A)** The results of GO enrichment in the TCGA cohort. “BP” stands for “biological process,” “CC” stands for “cellular component” and “MF” stands for “molecular function”. The abscissa represents the gene ratio. **(B)** The results of KEGG enrichment in the TCGA cohort. The abscissa represents the gene ratio.

The expression data of 375 cases of gastric cancer tissues and 32 normal gastric tissues, for a total of 407 cases, were downloaded from the TCGA database, together with the expression data, survival status, and clinical data of 435 cases of gastric cancer patients. After extracting the patient information including survival status and survival time among these 435 GC patients, and intersecting them with the 407 patients for which RNA-seq data were available, we obtained 368 GC patients that presented RNA-seq data and clinical data and used it as a training set to build the model. 121 pyroptosis-related genes were obtained from the human gene database. R-Package “Limma” was used to screen pyroptosis-related genes in gastric cancer. The screening criteria were logFC >0.5, FDR <0.05. The results showed that 51 genes were identified as differentially expressed in gastric cancer (Figure 2). GO analysis showed that these genes were mainly enriched in the basic biological processes (BP) of positive regulation of cytokine (Mirgayazova et al., 2019) production, cellular response to biotic stimulus, defense response to viruses, and pyroptosis (Figure 3A). KEGG analysis showed that these genes were mainly related to NOD−like receptor signaling pathway, apoptosis, and *Salmonella* infection (Figure 3B).

### PPI Network Analysis

**FIGURE 4:**
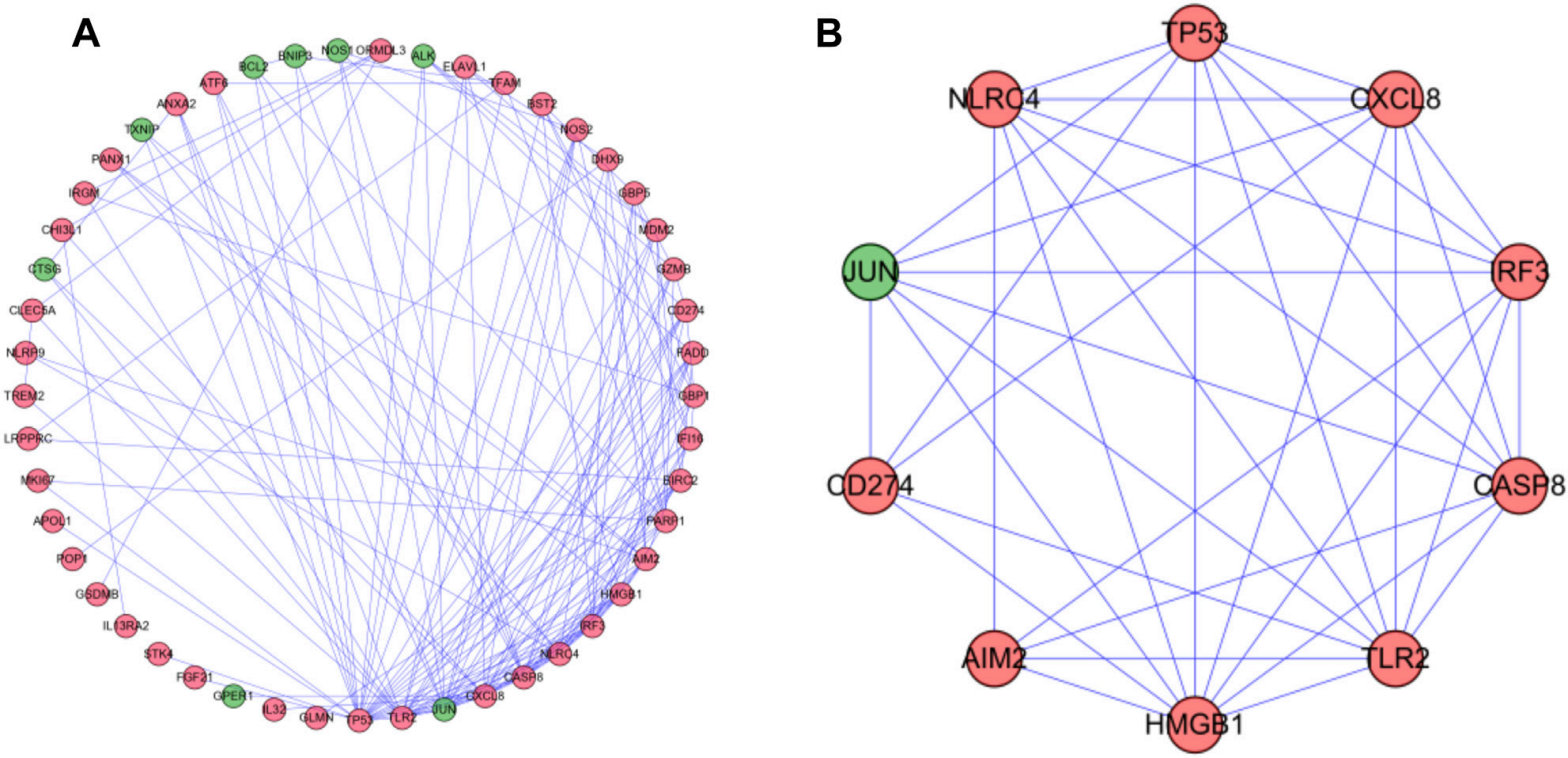
PPI network and modules analysis. **(A)** PPI network for DEGs. **(B)** Key module in PPI network. Red: upregulation; Green: downregulation.

To further understand the role of differential genes in the GC process, we use the STRING database and Cytoscape software to construct a PPI network, which is composed of 49 nodes and 155 edges (Figure 4A). Then, we used the MODE plugin Cytoscape to identify the key modules from the PPI network. The modules included nine up-regulated differentially expressed genes (DEGs) and one down-regulated DEGs (Figure 4B). Enrichment analysis showed that they were related to positive regulation of cytokine production, hepatitis, and NOD-like receptor signaling pathway.

### Construction and Verification of Prognostic Genes Related to Pyroptosis

**FIGURE 5:**
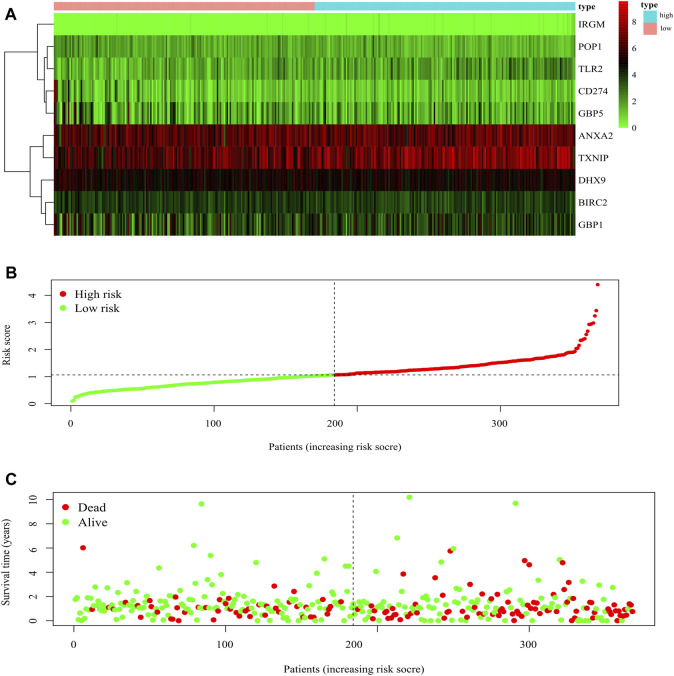
Characteristics of prognostic gene signatures. **(A)** The heatmap of the ten DEGs expression profiles in high- and low-risk GC patients. The abscissa represents risk types, the ordinate represents the gene. **(B)** Distribution of risk scores of high- and low-risk GC patients. The abscissa represents the patients (increasing risk score), the ordinate represents the risk score. **(C)** The scatter plot shows the correlation between survival time and risk score. The abscissa represents the patients (increasing risk score), the ordinate represents survival time (years).

The 51 differentially expressed genes screened above were included in univariate COX analysis, and 47 genes related to prognosis were screened (Supplementary Material S2). The 47 genes were included in multivariate Cox regression analysis to construct prognostic markers, and 10 genes (BIRC2, CD274, IRGM, ANXA2, GBP5, TXNIP, POP1, GBP1, DHX9, and TLR2) related to prognosis were obtained. The coefficients of each gene are shown in Table 1. The risk score was calculated as follows: risk score = (−0.400605791695688*BIRC2 expression) + (−0.323210285810829*CD274 expression) + (2.35259453336258*IRGM expression) + (0.267383737123388*ANXA2 expression) + (−0.252281344553063*GBP5 expression) + (0.142945074684017*TXNIP expression) + (−0.479593898864018*POP1 expression) + (0.301409058780011*GBP1 expression) + (0.539214896060124*DHX9 expression) + (0.295854165226579*TLR2 expression). The risk score of each gastric cancer patient was calculated according to the expression level of the ten genes, and the patients were divided into high-risk and low-risk according to the median risk score. The gene expression profiles of the high-risk group and low-risk group are shown by the heatmap (Figure 5A). Figure 5B shows the risk score distribution of patients with gastric cancer, which increases gradually from left to right and divides the patients into two groups. Figure 5C shows the distribution of survival status and survival time of patients with different risk scores.

**TABLE 1:** Genes included in the prognostic gene signature.

Gene symbol	Coefficient	HR	*p* Value
BIRC2	−0.400605792	0.669914095	0.099458
CD274	−0.323210286	0.723821629	0.140321
IRGM	2.352594533	10.51281021	0.001412
ANXA2	0.267383737	1.306541719	0.036579
GBP5	−0.252281345	0.777026095	0.107473
TXNIP	0.142945075	1.153666434	0.116587
POP1	−0.479593899	0.619034731	0.057659
GBP1	0.301409059	1.351762179	0.074925
DHX9	0.539214896	1.714660147	0.050588
TLR2	0.295854165	1.344274101	0.03618

### Validation of the 10-Gene Signature

**FIGURE 6:**
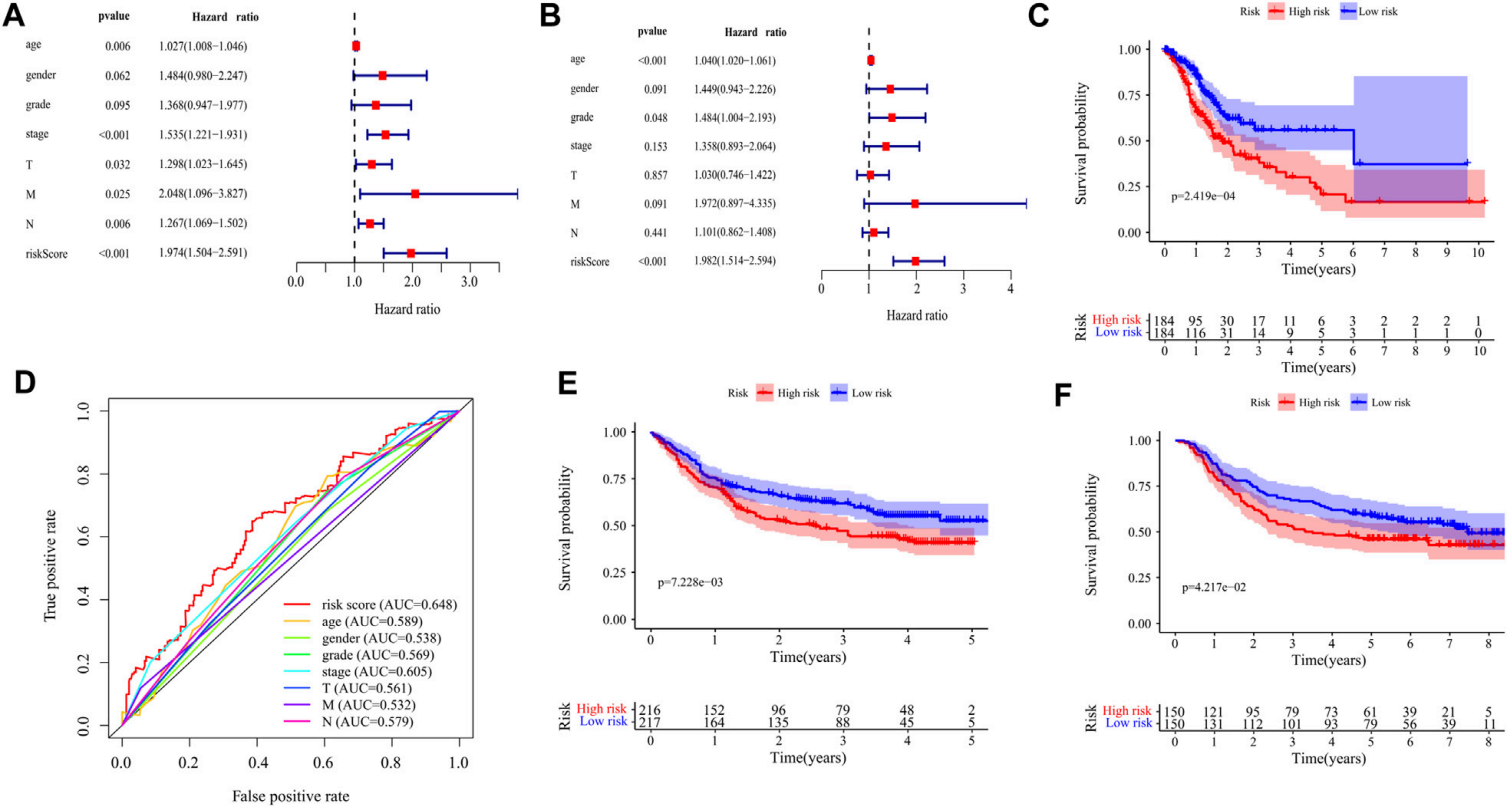
Validation of the prognostic signature of ten DEGs. **(A)** The Forest plot reflects the univariate Cox analysis of the relationship between the clinical features, risk score, and OS of GC patients. Both stage and risk score significantly affect the prognosis of GC patients (*p* < 0.001). **(B)** The Forest plot reflects the multivariate Cox analysis of the relationship between the clinical features, risk score, and OS of GC patients. Age and risk score are independent prognostic risk factors for GC (*p* < 0.001). **(C)** The Kaplan-Meier Survival curve shows that the OS of high-risk GC patients is significantly lower than that of low-risk patients. The abscissa represents time (years), the ordinate represents survival probability. **(D)** The 1-year time-dependent ROC curve shows that the prediction accuracy of the risk score is higher than other clinical features (AUC = 0.648). The abscissa represents false positive rate, the ordinate represents true positive rate. **(E)** Kaplan-Meier Survival Curve of patients with GC in high-risk and low-risk groups in GSE84437 (*p* = 7.228e−03). The abscissa represents time (years), the ordinate represents survival probability. **(F)** Kaplan-Meier Survival Curve of patients with GC in high-risk and low-risk groups in GSE66229 (*p* = 4.217e−02). The abscissa represents time (years), the ordinate represents survival probability.

The prognostic value of the risk score was evaluated by univariate and multivariate analysis. Univariate analysis showed that there was a significant correlation between risk score and overall survival (OS) (HR = 1.974,95%CI = 1.504–2.591, *p* < 0.001) (Figure 6A). Multivariate analysis showed that risk score was an independent prognostic index (HR = 1.982,95%CI = 1.514–2.594, *p* < 0.001) (Figure 6B). Kaplan-Meier cumulative curve shows that patients with a low-risk score have a longer survival time than patients with a high-risk score (Figure 6C). The AUC of risk score was higher than that of sex, age, pathological grade, and TNM stage, which proved that the Cox model was better than other single indexes in predicting the prognosis (Figure 6D). To verify the predictive value of this prognostic model, we used the same formula to calculate the risk scores of patients in the GSE84437 and GSE66229 datasets. The OS of the high-risk group was significantly lower than that of the low-risk group (GSE84437: *p* = 7.228e−03; GSE66229: *p* = 4.217e−02), which is consistent with the results in the TCGA cohort (Figures 6E,F).

### Expression and Alteration of the Ten Prognosis-Related RBP Genes

**FIGURE 7:**
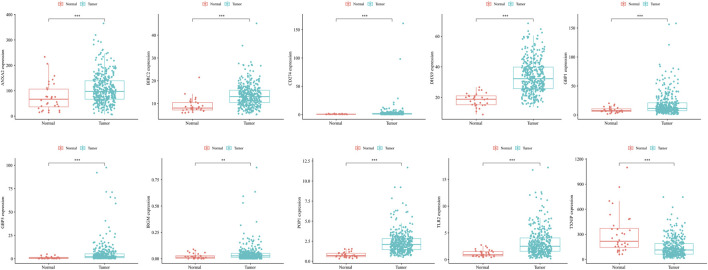
The ten genes were differentially expressed between normal samples and tumor samples (* = *p* < 0.05, ** = *p* < 0.01, *** = *p* < 0.001). The abscissa represents tissues, the ordinate represents gene expression.

**FIGURE 8:**
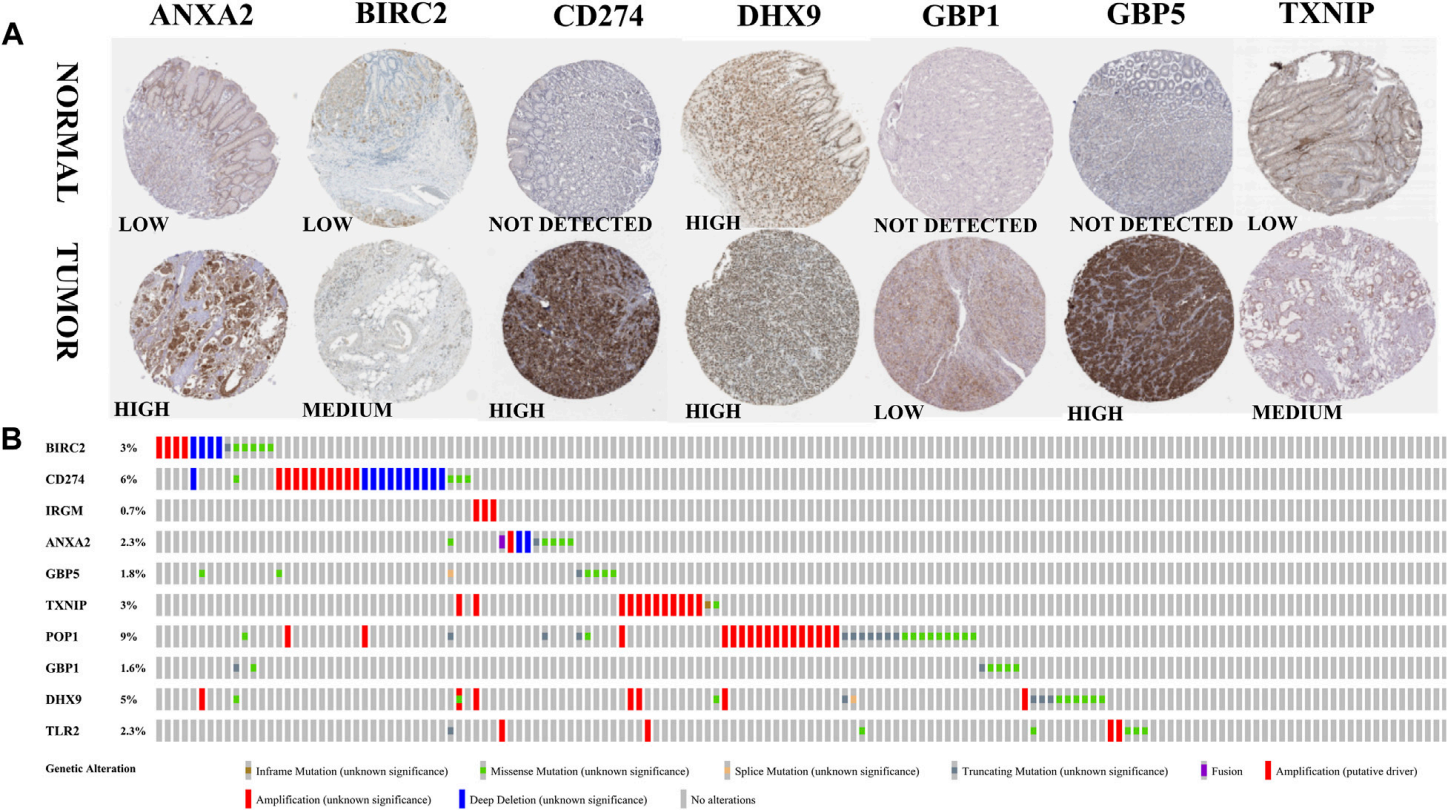
Expression and alteration of the ten prognosis-related RBP genes. **(A)** The representative protein expression of the seven genes in GC and normal tissue. Data were from the Human Protein Atlas (http://www.proteinatlas.org) online database. **(B)** The expression alteration profiles of the ten genes in the TCGA GC RNA-seq dataset.

The expression of model genes in GC was observed by analyzing the difference of model genes between normal samples and tumor samples (Figure 7). The ten genes were differentially expressed between normal samples and tumor samples (* = *p* < 0.05, ** = *p* < 0.01, *** = *p* < 0.001). We further analyzed the expression of these model genes through the HPA database. Figure 8A shows the immunohistochemical results of seven key RBPs in GC and normal tissues. IRGM, POP1, and TLR2 are not included in the database. By using the cBioPortal online database, we found that the main alteration of 10 RBP genes identified in CG patients was amplification (Figure 8B).

### The Correlation Between the Clinical Features and Risk Score of GC Patients

**FIGURE 9:**
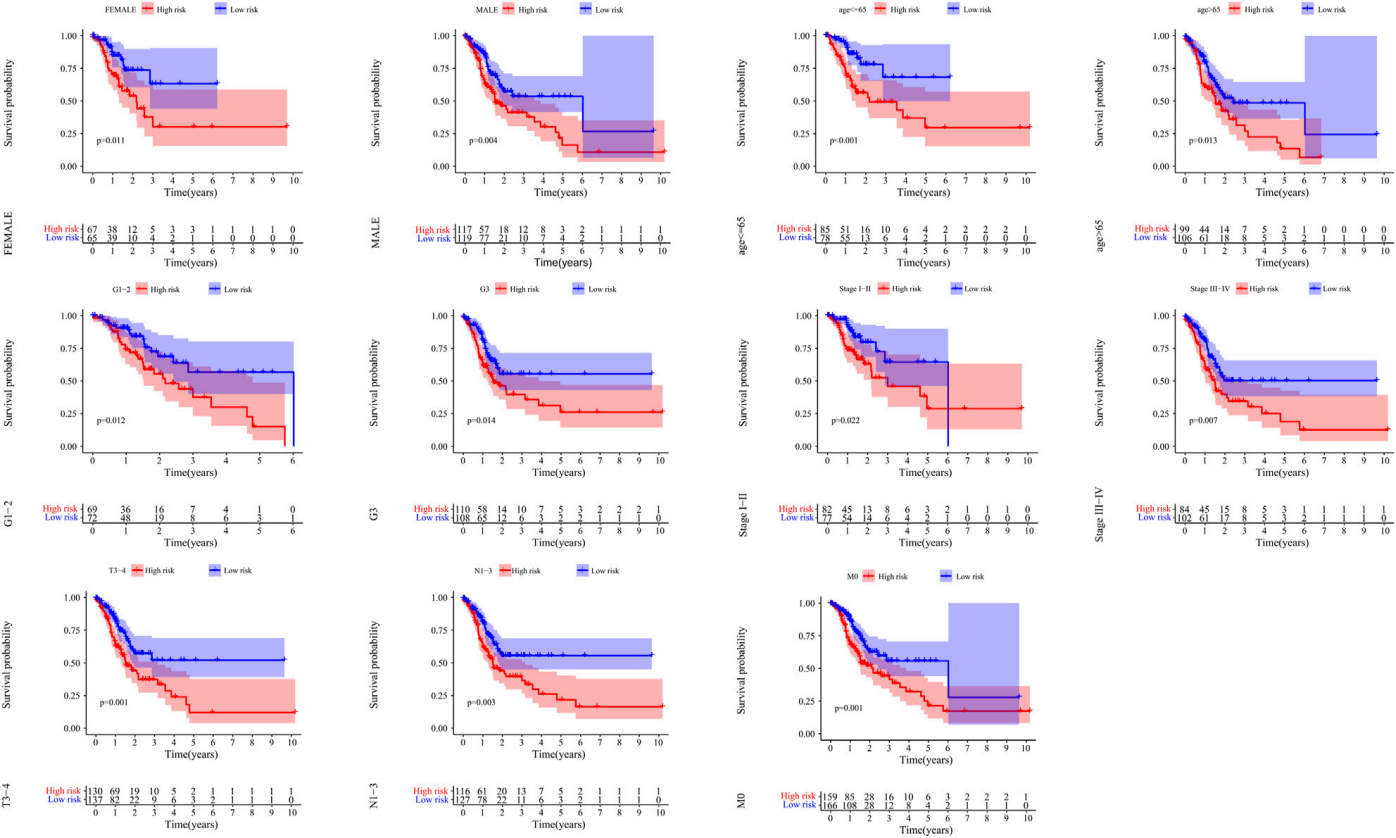
The correlation between the clinical features and risk score of GC patients. The abscissa represents time (years), the ordinate represents survival probability.

Kaplan-Meier curves showed that patients with low risk had a better prognosis in >65 years old, ≤ 65 years old, male, female, G1-2, G3, M0, N1-3, StageI-II, StageIII-IV and T3-4 (*p* < 0.05) (Figure 9).

### Construction and Verification of Nomogram

**FIGURE 10:**
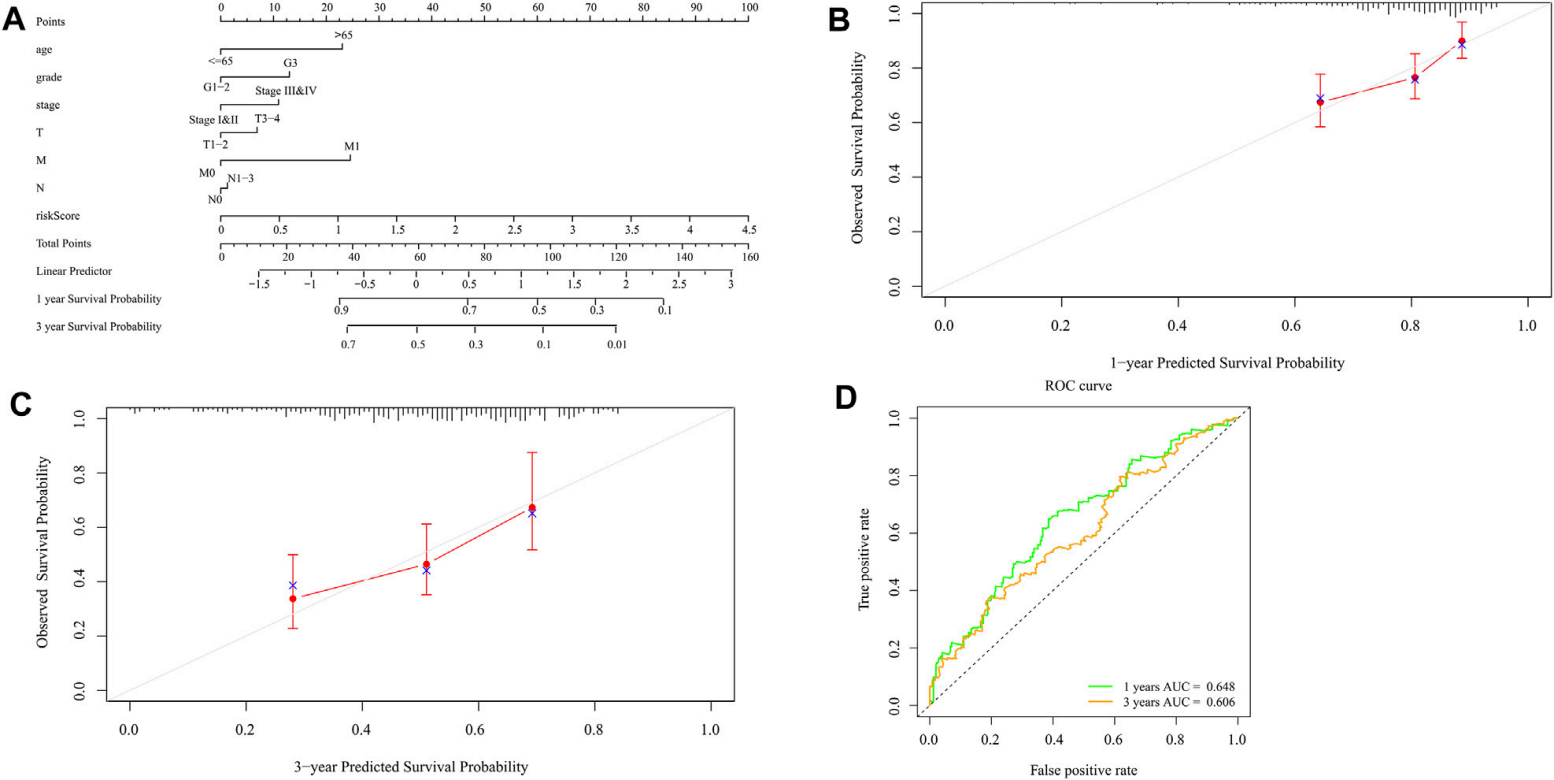
Construction and validation of the nomogram. **(A)** Scores of each item of GC patients were calculated according to the nomogram, and the total scores obtained after addition can predict the 1- and 3-year survival probability. **(B,C)** The 1- and 3-year calibration curves of the nomogram **(D)** The ROC curves of 1-and 3-year nomogram (AUC = 0.648 for 1 year, AUC = 0.606 for 3 years). The abscissa represents false positive rate, the ordinate represents true positive rate.

Nomograms can be used to help clinical interpretation of predictive signals, and can easily determine the survival rate of patients with gastric cancer. By combining the characteristics of ten pyroptosis-related prognostic genes with clinicopathological features, a nomogram for predicting individual survival probability was established (Figure 10A), and the possibility of 1-year and 3-year OS was predicted. When the calibration curve is closer to the diagonal, it is proved that the prediction result is more accurate (Figures 10B,C). The 1-year and 3-year ROC curves (Figure 10D) also show that the predictive ability of the nomogram is good (1-year AUC = 0.648,3-year AUC = 0.606).

### Analysis of Immune Cell Infiltration

**FIGURE 11:**
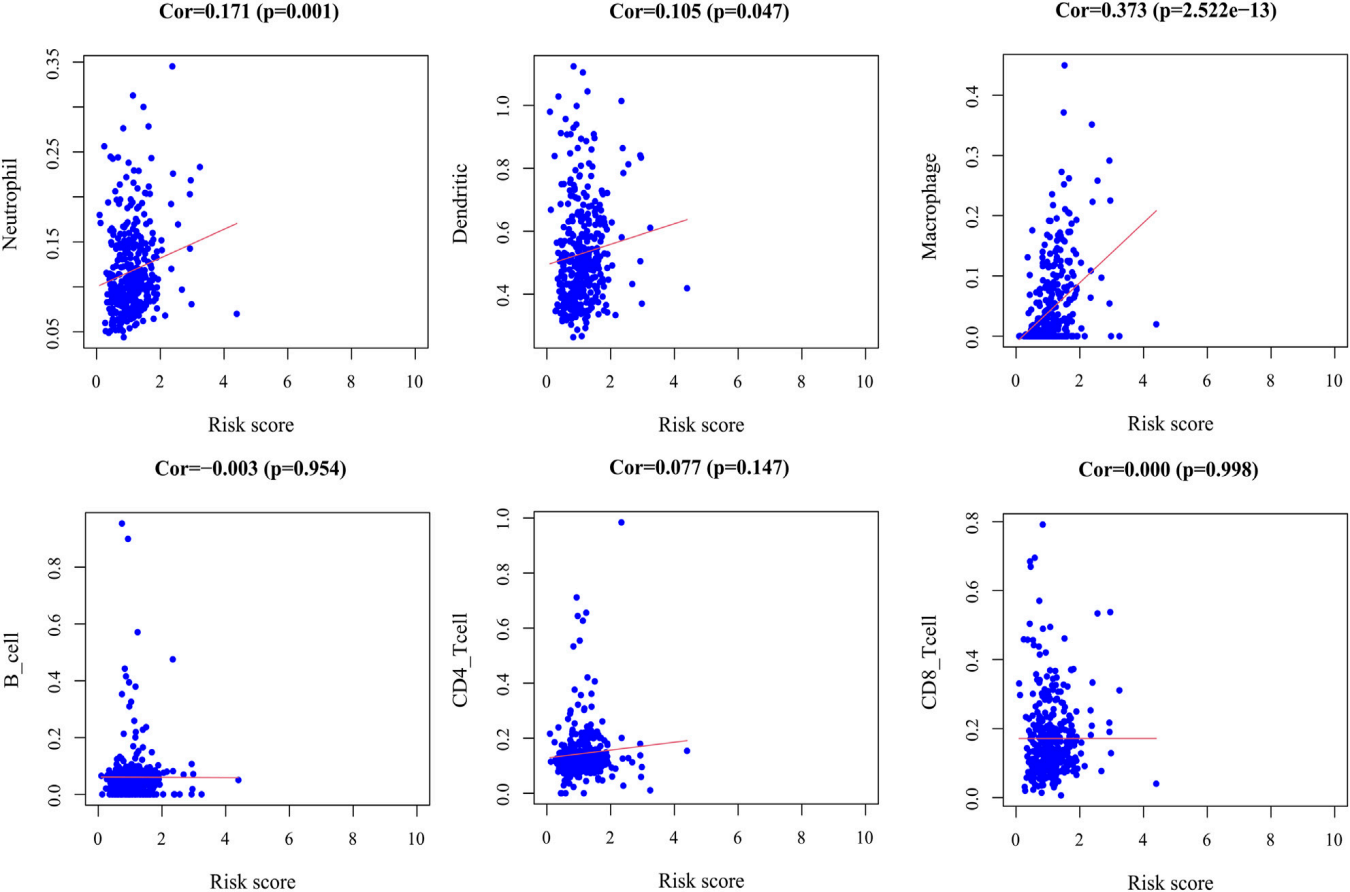
Correlation plot between risk score and immune cells infiltration. The abscissa represents risk score, the ordinate represents infiltration abundances of immune cells.

By exploring the relationship between the risk score model and immune cell infiltration, we found that dendritic cells, macrophages, and neutrophils were positively correlated with the risk score. However, there was no significant correlation between B cells, CD8+T cells, and CD4+T cells and the risk score (Figure 11).

## Discussion

The prognosis of gastric cancer, one of the most common malignant tumors in the world, is still not optimistic. Surgery is the more reliable treatment at present, but further treatment of patients after surgery and conservative treatment of advanced patients have limited benefits. Therefore, to achieve early diagnosis and find treatment targets, it is particularly important to explore the pathogenesis of gastric cancer and establish effective prognostic criteria, which may help patients to develop personalized treatment plans.

In this study, we analyzed the transcriptome information and clinical data of GC patients in the TCGA database. Univariate Cox analysis showed that 47 pyroptosis genes were associated with prognosis. Through multivariate Cox analysis, we established a prognostic model based on ten pyroptosis genes, including BIRC2, CD274, IRGM, ANXA2, GBP5, TXNIP, POP1, GBP1, DHX9, and TLR2. The risk score of each patient can be calculated from the mRNA expression level and risk coefficient of these ten genes. This is an independent risk factor that can affect the prognosis and can predict that the high-risk CG patients have a worse prognosis than the low-risk patients. Through the ROC curve analysis of the survival rate of patients with GC, it is found that the prognostic index has good sensitivity and specificity (AUC = 0.648), which can be used as a reliable predictor of the prognosis of patients with GC. Moreover, we could successfully verify our results in two independent datasets, GSE84437 and GSE66229. We also constructed a nomogram to predict the 1-and 3-year survival rates of patients for clinical application and to obtain more accurate prediction results. The nomogram has better prediction accuracy than the correction curve and ROC curve. Predicted results and the actual results are in good agreement.

BIRC2 is a closely related member of the inhibitor of apoptosis (IAP) family, which plays a key role in nuclear factor JB (NFjB) signal transduction and apoptosis (Yamato et al., 2015). BIRC2 is an effective negative regulator of LTR-dependent HIV-1 transcription (Pache et al., 2015). When its expression is absent, it can inhibit the growth of breast cancer or melanoma through an immune-mediated mechanism (Samanta et al., 2020). On the contrary, the overexpression of BIRC2 promotes the metastasis of gastric cancer cells (Chen et al., 2020a). CD274 (Hou et al., 2020a) is a key molecule of tumor immune checkpoint mechanisms, is one of the main targets of immunotherapy (Fabrizio et al., 2018), and plays an important role in tumor immune escape (Huang et al., 2019). Cancer cells expressing CD274 may affect regulatory T cells in the tumor microenvironment (Masugi et al., 2017). PD-L1 switches TNFα-induced apoptosis to pyroptosis in cancer cells, resulting in tumor necrosis (Hou et al., 2020b). IRGM is a human protein of the immune-associated GTPase family that promotes autophagy during inflammation and infection (Song et al., 2015). It has been reported that IRGM plays an important role in non-small cell lung cancer (Wang et al., 2018) and liver cancer by regulating autophagy (Chen et al., 2021). In addition, IRGM interacts with NLRP3 and ASC and blocks the assembly of inflammatory bodies by blocking the oligomerization of NLRP3 and ASC (Mehto et al., 2019). ANXA2 is a 36 kDa calcium-dependent phospholipid-binding cytoskeletal protein. When ANXA2 is silenced, the ability of proliferation, invasion, and migration of gastric cancer cells is weakened (Xie et al., 2019), but when it is overexpressed, it can promote the migration, invasion, and metastasis of esophageal cancer cells *in vitro* and *in vivo* by activating the MYC-HIF1a-VEGF cascade pathway (Ma et al., 2018). GBP5 and GBP1 are interferon-inducible GTPases belonging to the guanylate binding protein (GBP) family, which can promote antibacterial immunity and cell death. They support the activation of caspase-1-containing inflammasome complexes or caspase-4, which triggers pyroptosis (Fisch et al., 2019). Studies by Jing Zhao et al. (2019) have shown that GBP1 can promote survival or carcinogenesis in prostate cancer. TXNIP is the only known α-arrestin protein family that binds to Trx (Schröder et al., 2020). The expression of TXNIP in tumors is very low, and it may play an inhibitory role in many kinds of cancers such as liver cancer, breast cancer and lung cancer (Chen et al., 2020b). POP1 is an intact membrane protein that regulates the formation of tight junctions (Williams et al., 2011). It inhibits the assembly of ASC-dependent inflammatory bodies by preventing the nucleation of inflammatory bodies, thereby interfering with the activation of caspase-1, the release of IL-1b and IL-18, pyroptosis and the release of ASC particles (de Almeida et al., 2015). DHX9 is a member of RNA helicase DExH subgroup, which plays an important role in several aspects of RNA metabolism (Palombo et al., 2020). The expression of DHX9 is up-regulated in cervical cancer tissue, which promotes the movement and angiogenesis of cervical cancer cells. Moreover, DHX9 plays an important role in promoting the metastasis of colorectal cancer (Hou et al., 2021). TLR2 belongs to the Toll-like receptor family and is a key regulator of innate and acquired immune responses (Cao et al., 2019).

We found that dendritic cells, macrophages, and neutrophils infiltration into CG tumors were positively correlated with the risk score. Macrophages create an inflammatory environment that is mutagenic and promotes growth at the beginning of tumor formation. With tumor development, macrophages stimulate angiogenesis, enhance the migration and invasion of tumor cells, and inhibit anti-tumor immunity (Qian and Pollard, 2010). It has been confirmed that macrophages play an important role in the occurrence and development of gastric cancer (Chen et al., 2017; Zheng et al., 2017; Eissmann et al., 2019; Gambardella et al., 2020). Dendritic cells are powerful antigen-presenting cells that can stimulate immature resting T cells and initiate the initial immune response (Wu et al., 2004). The fusion vaccine of allogeneic dendritic cells and tumor cells can be used to enhance the effect of immunotherapy in patients with gastric cancer (Li et al., 2015). Neutrophils are the first responders to inflammation and infection. Tumor-associated neutrophils can promote tumor inflammation by promoting angiogenesis, extracellular matrix remodeling, metastasis, and immunosuppression (Mollinedo, 2019). It has been confirmed that tumor-associated neutrophils can promote the progression and metastasis of gastric cancer in many ways (Jaillon et al., 2020). Shao et al. (2021) established the gastric cancer score related to pyroptosis, which proved its significant correlation with the immune microenvironment, and further confirmed our research results. It provides support for the development of immunotherapy strategies related to pyroptosis in the future.

This study has some limitations. The conclusion of this study is based on bioinformatic analyses and lacks further verification *in vivo* and *in vitro*. The samples come from a retrospective study, so it is necessary to conduct a more in-depth prospective clinical study of the signature and nomogram.

In summary, we have established a new prognostic model for gastric cancer and pyroptosis. The score generated by the risk signature of the markers is an independent risk factor for predicting OS. According to the established nomogram, the 1-and 3-year survival rate of patients with gastric cancer can be predicted, which provides a reference for the formulation of personalized treatment for patients with gastric cancer, and provides an important basis for further study of the relationship between gastric cancer and pyroptosis.

## Data Availability

The datasets analyzed for this study can be found in The Cancer Genome Atlas (https://portal.gdc.cancer.gov/) and THE HUMAN GENE DATABASE (https://www.genecards.org/).
